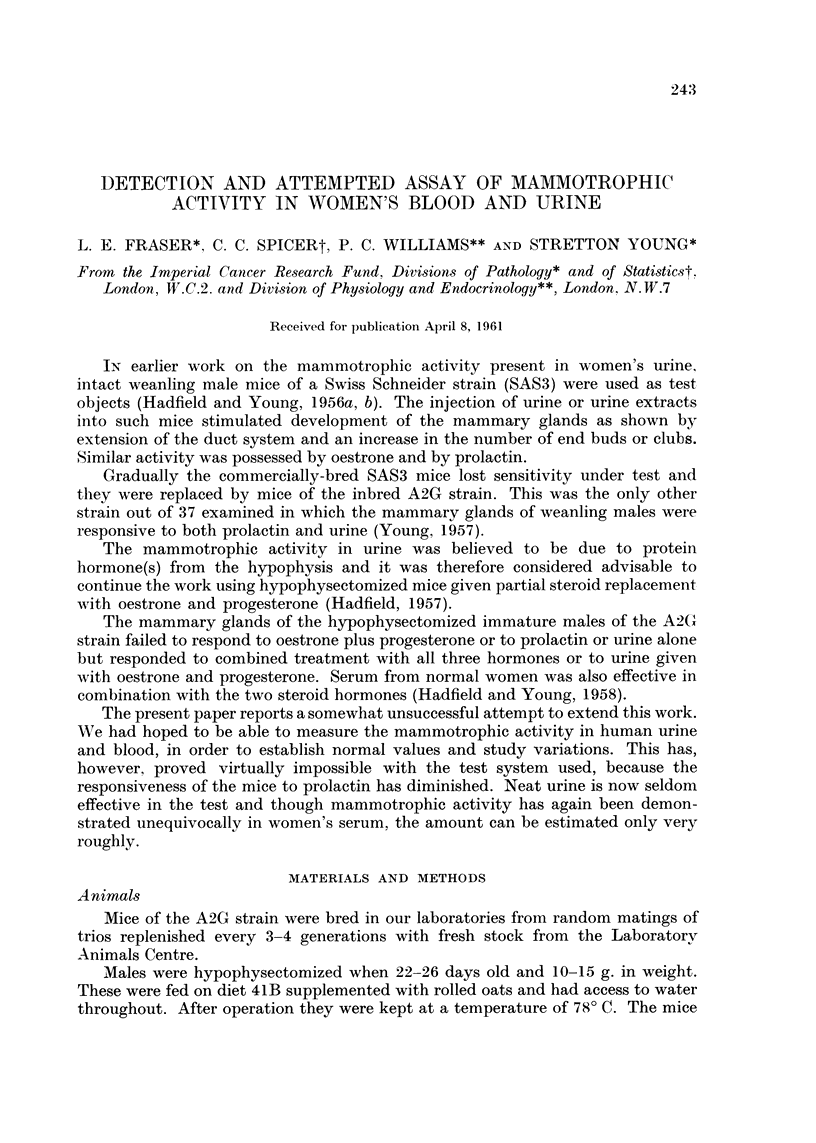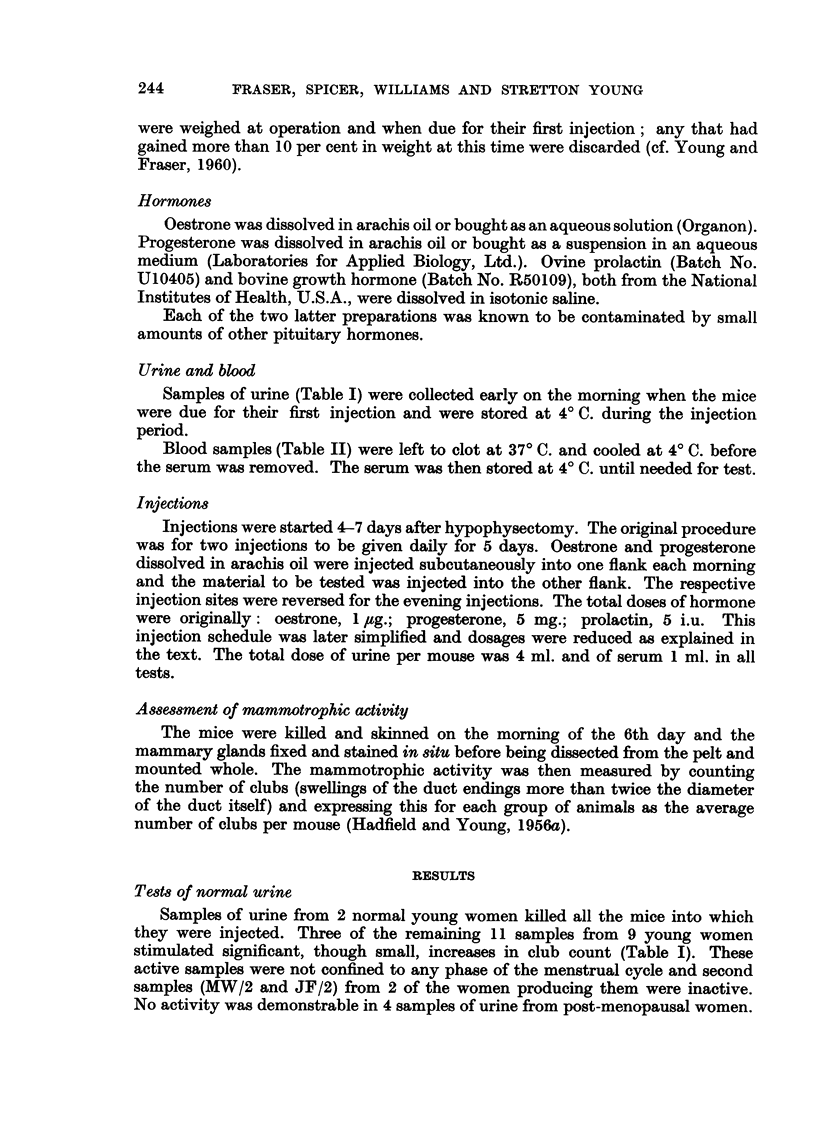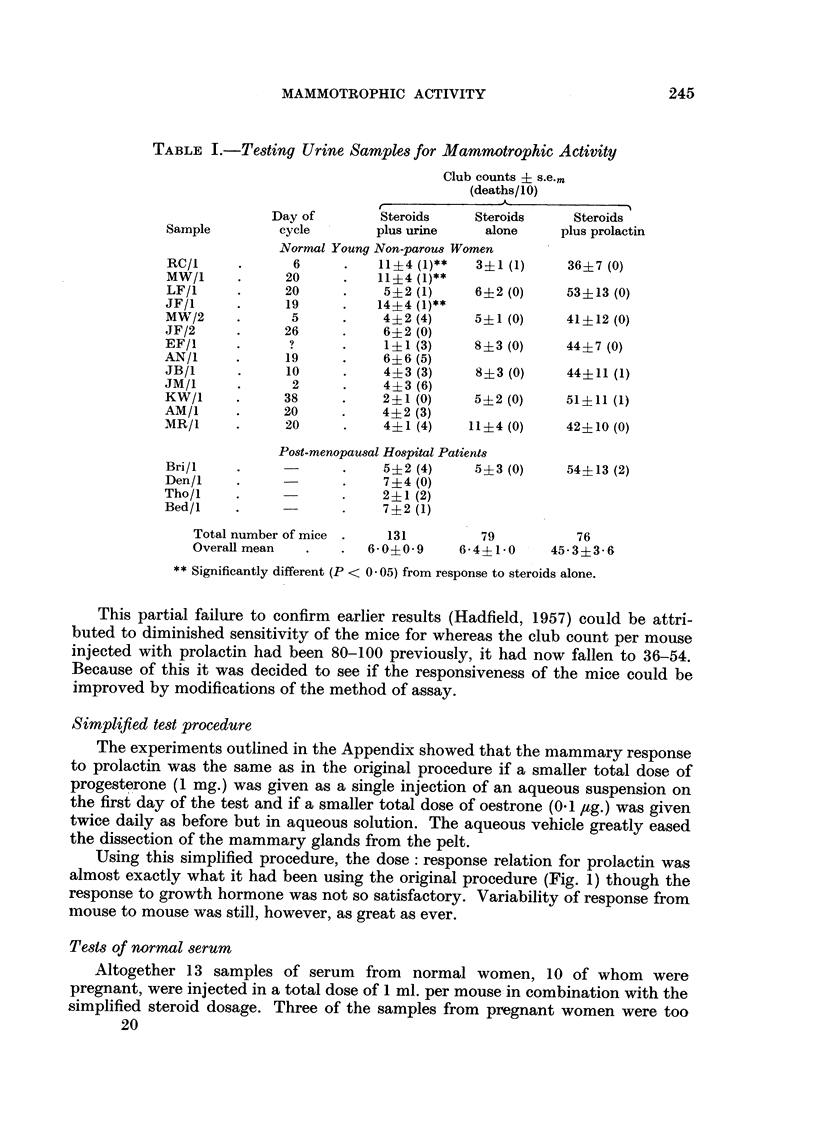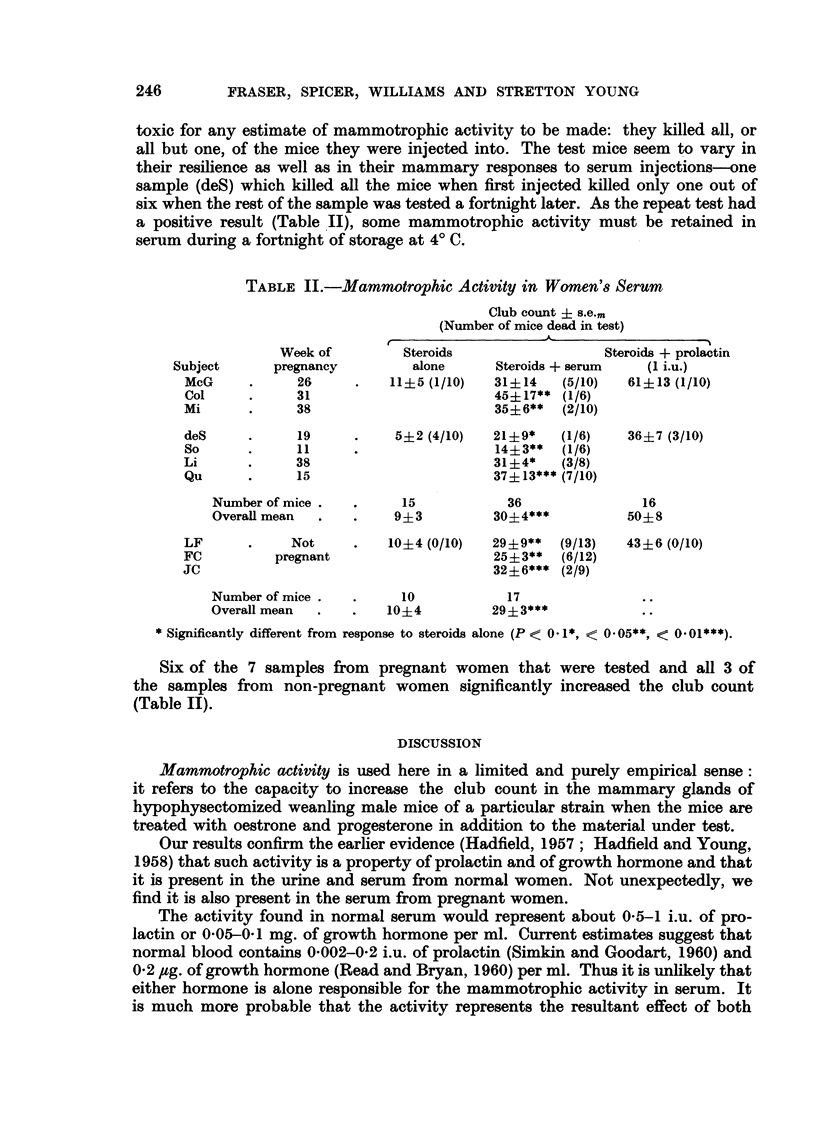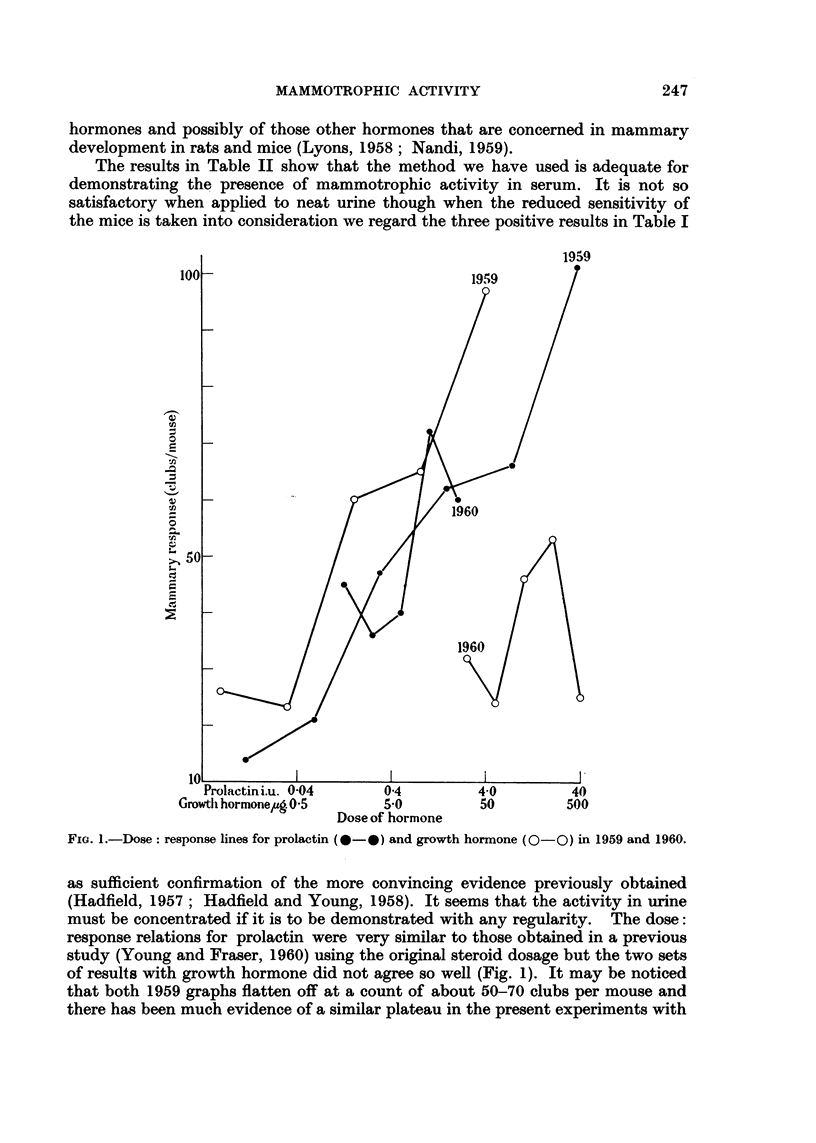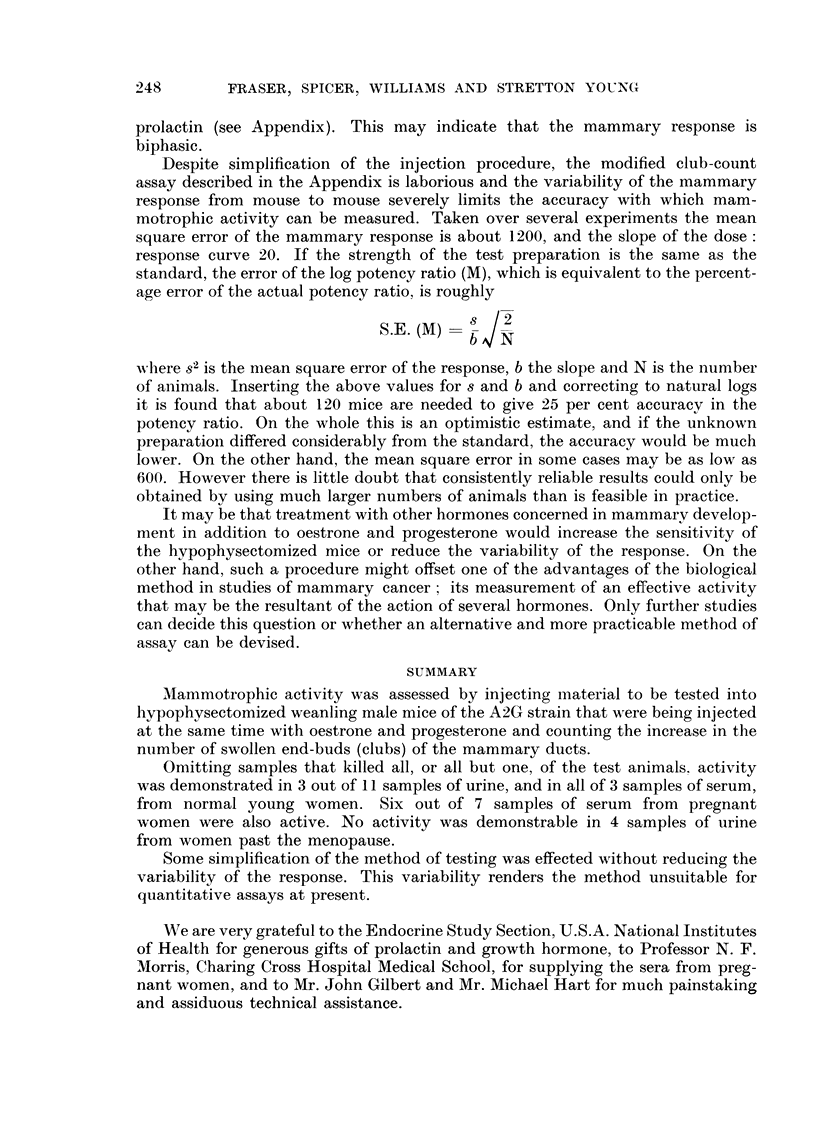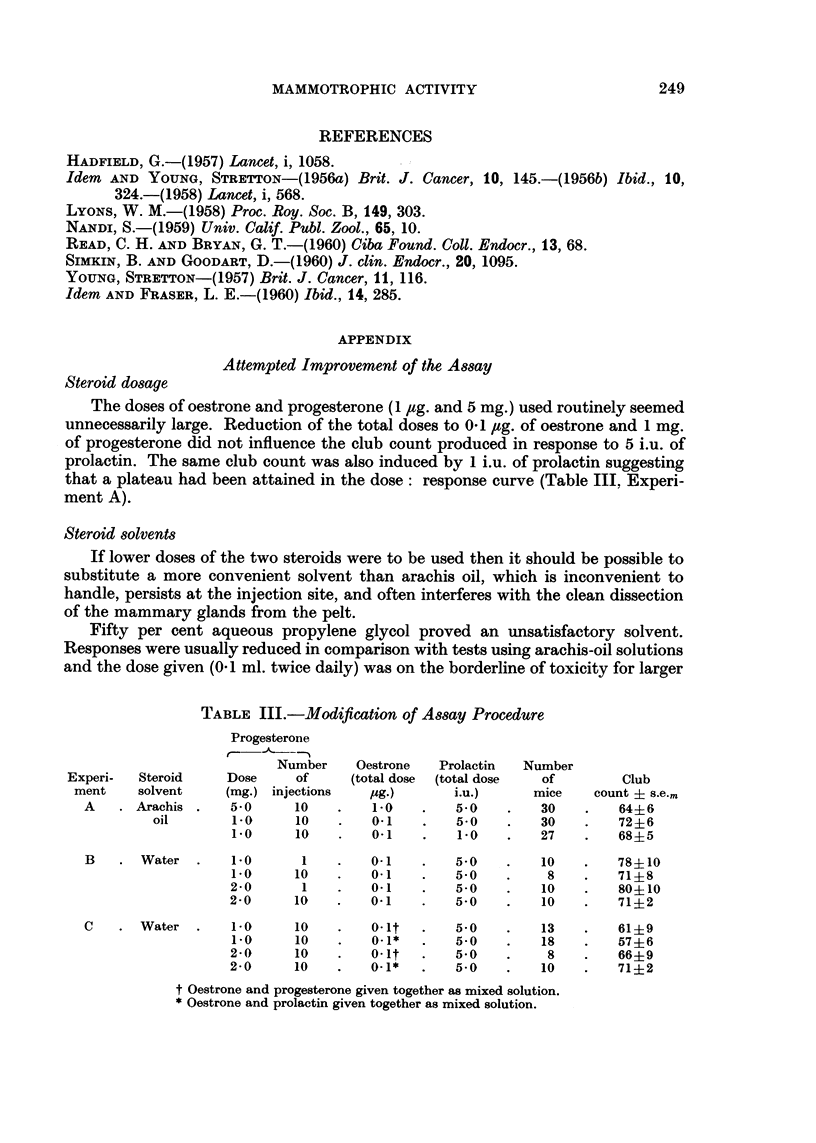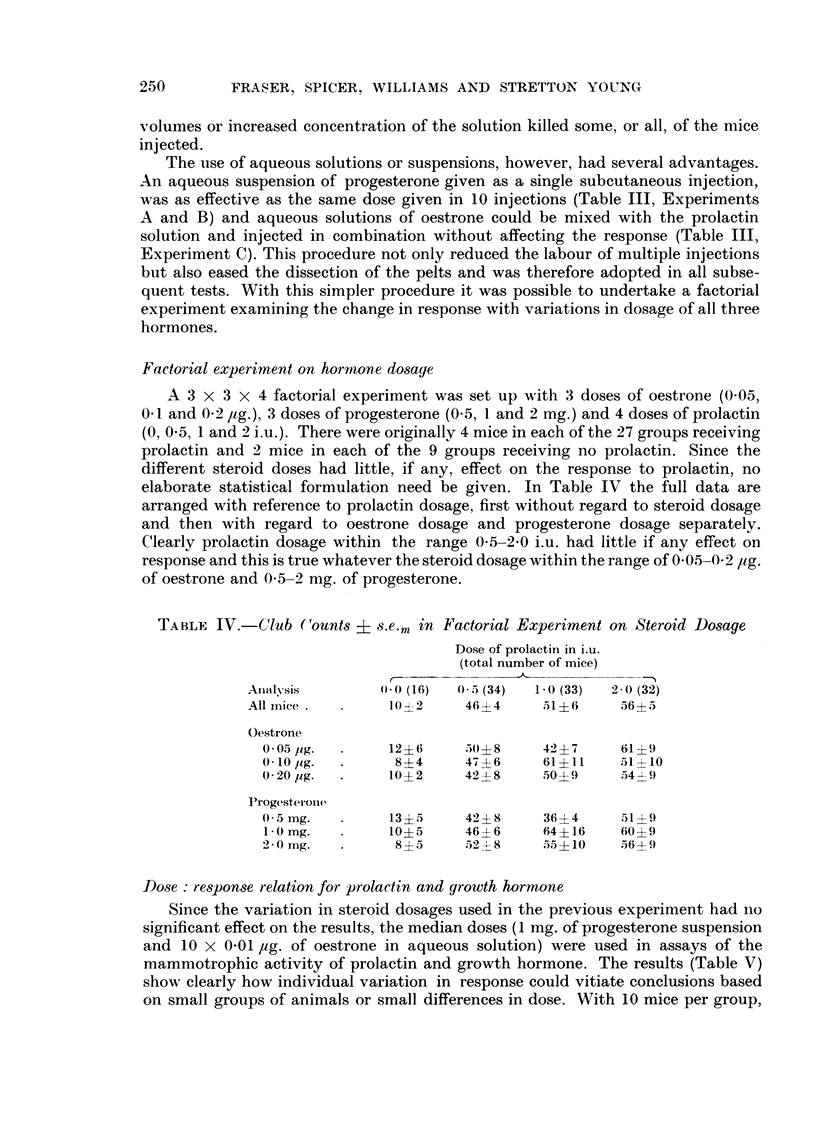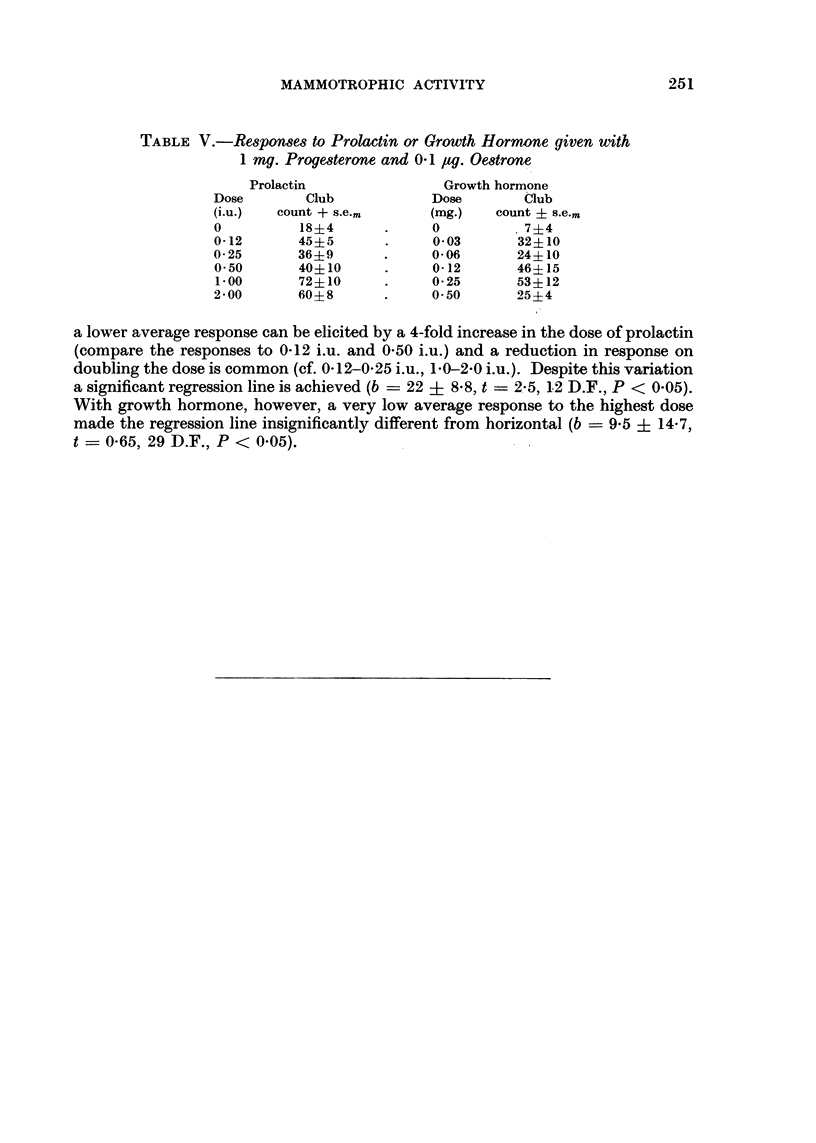# Detection and Attempted Assay of Mammotrophic Activity in Women's Blood and Urine

**DOI:** 10.1038/bjc.1961.30

**Published:** 1961-06

**Authors:** L. E. Fraser, C. C. Spicer, P. C. Williams, Stretton Young


### DETECTION AND ATTEMPTED ASSAY OF MAMMOTROPHIC ACTIVITY IN WOMEN'S BLOOD AND URINE

L. E. FRASER*, C. C. SPICER†, P. C. WILLIAMS** AND STRETTON YOUNG*

*From the Imperial Cancer Research Fund, Divisions of Pathology* and of Statistics†, London, W.C.2. and Division of Physiology and Endocrinology**, London, N.W.7*

Received for publication April 8, 1961

IN earlier work on the mammotrophic activity present in women's urine, intact weanling male mice of a Swiss Schneider strain (SAS3) were used as test objects (Hadfield and Young, 1956*a*, *b*). The injection of urine or urine extracts into such mice stimulated development of the mammary glands as shown by extension of the duct system and an increase in the number of end buds or clubs. Similar activity was possessed by oestrone and by prolactin.

Gradually the commercially-bred SAS3 mice lost sensitivity under test and they were replaced by mice of the inbred A2G strain. This was the only other strain out of 37 examined in which the mammary glands of weanling males were responsive to both prolactin and urine (Young, 1957).

The mammotrophic activity in urine was believed to be due to protein hormone(s) from the hypophysis and it was therefore considered advisable to continue the work using hypophysectomized mice given partial steroid replacement with oestrone and progesterone (Hadfield, 1957).

The mammary glands of the hypophysectomized immature males of the A2G strain failed to respond to oestrone plus progesterone or to prolactin or urine alone but responded to combined treatment with all three hormones or to urine given with oestrone and progesterone. Serum from normal women was also effective in combination with the two steroid hormones (Hadfield and Young, 1958).

The present paper reports a somewhat unsuccessful attempt to extend this work. We had hoped to be able to measure the mammotrophic activity in human urine and blood, in order to establish normal values and study variations. This has, however, proved virtually impossible with the test system used, because the responsiveness of the mice to prolactin has diminished. Neat urine is now seldom effective in the test and though mammotrophic activity has again been demonstrated unequivocally in women's serum, the amount can be estimated only very roughly.

### MATERIALS AND METHODS

### *Animals*

Mice of the A2G strain were bred in our laboratories from random matings of trios replenished every 3–4 generations with fresh stock from the Laboratory Animals Centre.

Males were hypophysectomized when 22–26 days old and 10–15 g. in weight. These were fed on diet 41B supplemented with rolled oats and had access to water throughout. After operation they were kept at a temperature of 78° C. The mice were weighed at operation and when due for their first injection; any that had gained more than 10 per cent in weight at this time were discarded (cf. Young and Fraser, 1960).

*Hormones*

Oestrone was dissolved in arachis oil or bought as an aqueous solution (Organon). Progesterone was dissolved in arachis oil or bought as a suspension in an aqueous medium (Laboratories for Applied Biology, Ltd.). Ovine prolactin (Batch No. U10405) and bovine growth hormone (Batch No. R50109), both from the National Institutes of Health, U.S.A., were dissolved in isotonic saline.

Each of the two latter preparations was known to be contaminated by small amounts of other pituitary hormones.

*Urine and blood*

Samples of urine (Table I) were collected early on the morning when the mice were due for their first injection and were stored at 4° C. during the injection period.

Blood samples (Table II) were left to clot at 37° C. and cooled at 4° C. before the serum was removed. The serum was then stored at 4° C. until needed for test.

*Injections*

Injections were started 4–7 days after hypophysectomy. The original procedure was for two injections to be given daily for 5 days. Oestrone and progesterone dissolved in arachis oil were injected subcutaneously into one flank each morning and the material to be tested was injected into the other flank. The respective injection sites were reversed for the evening injections. The total doses of hormone were originally: oestrone, 1 $\mu$g.; progesterone, 5 mg.; prolactin, 5 i.u. This injection schedule was later simplified and dosages were reduced as explained in the text. The total dose of urine per mouse was 4 ml. and of serum 1 ml. in all tests.

*Assessment of mammotrophic activity*

The mice were killed and skinned on the morning of the 6th day and the mammary glands fixed and stained *in situ* before being dissected from the pelt and mounted whole. The mammotrophic activity was then measured by counting the number of clubs (swellings of the duct endings more than twice the diameter of the duct itself) and expressing this for each group of animals as the average number of clubs per mouse (Hadfield and Young, 1956*a*).

### RESULTS

*Tests of normal urine*

Samples of urine from 2 normal young women killed all the mice into which they were injected. Three of the remaining 11 samples from 9 young women stimulated significant, though small, increases in club count (Table I). These active samples were not confined to any phase of the menstrual cycle and second samples (MW/2 and JF/2) from 2 of the women producing them were inactive. No activity was demonstrable in 4 samples of urine from post-menopausal women.

TABLE I.—*Testing Urine Samples for Mammotrophic Activity*

| Sample | Day of cycle | Club counts ± s.e.$_m$ (deaths/10) Steroids plus urine | Steroids alone | Steroids plus prolactin |
|---|---|---|---|---|
| | | *Normal Young Non-parous Women* | | |
| RC/1 | 6 | 11±4 (1)** | 3±1 (1) | 36±7 (0) |
| MW/1 | 20 | 11±4 (1)** | | |
| LF/1 | 20 | 5±2 (1) | 6±2 (0) | 53±13 (0) |
| JF/1 | 19 | 14±4 (1)** | | |
| MW/2 | 5 | 4±2 (4) | 5±1 (0) | 41±12 (0) |
| JF/2 | 26 | 6±2 (0) | | |
| EF/1 | ? | 1±1 (3) | 8±3 (0) | 44±7 (0) |
| AN/1 | 19 | 6±6 (5) | | |
| JB/1 | 10 | 4±3 (3) | 8±3 (0) | 44±11 (1) |
| JM/1 | 2 | 4±3 (6) | | |
| KW/1 | 38 | 2±1 (0) | 5±2 (0) | 51±11 (1) |
| AM/1 | 20 | 4±2 (3) | | |
| MR/1 | 20 | 4±1 (4) | 11±4 (0) | 42±10 (0) |
| | | *Post-menopausal Hospital Patients* | | |
| Bri/1 | — | 5±2 (4) | 5±3 (0) | 54±13 (2) |
| Den/1 | — | 7±4 (0) | | |
| Tho/1 | — | 2±1 (2) | | |
| Bed/1 | — | 7±2 (1) | | |
| Total number of mice | | 131 | 79 | 76 |
| Overall mean | | 6·0±0·9 | 6·4±1·0 | 45·3±3·6 |

** Significantly different ($P < 0 \cdot 05$) from response to steroids alone.

This partial failure to confirm earlier results (Hadfield, 1957) could be attributed to diminished sensitivity of the mice for whereas the club count per mouse injected with prolactin had been 80–100 previously, it had now fallen to 36–54. Because of this it was decided to see if the responsiveness of the mice could be improved by modifications of the method of assay.

*Simplified test procedure*

The experiments outlined in the Appendix showed that the mammary response to prolactin was the same as in the original procedure if a smaller total dose of progesterone (1 mg.) was given as a single injection of an aqueous suspension on the first day of the test and if a smaller total dose of oestrone (0·1 $\mu$g.) was given twice daily as before but in aqueous solution. The aqueous vehicle greatly eased the dissection of the mammary glands from the pelt.

Using this simplified procedure, the dose : response relation for prolactin was almost exactly what it had been using the original procedure (Fig. 1) though the response to growth hormone was not so satisfactory. Variability of response from mouse to mouse was still, however, as great as ever.

*Tests of normal serum*

Altogether 13 samples of serum from normal women, 10 of whom were pregnant, were injected in a total dose of 1 ml. per mouse in combination with the simplified steroid dosage. Three of the samples from pregnant women were too toxic for any estimate of mammotrophic activity to be made: they killed all, or all but one, of the mice they were injected into. The test mice seem to vary in their resilience as well as in their mammary responses to serum injections—one sample (deS) which killed all the mice when first injected killed only one out of six when the rest of the sample was tested a fortnight later. As the repeat test had a positive result (Table II), some mammotrophic activity must be retained in serum during a fortnight of storage at 4° C.

TABLE II.—*Mammotrophic Activity in Women's Serum*

| Subject | Week of pregnancy | Club count ± s.e.$_m$ (Number of mice dead in test): Steroids alone | Steroids + serum | Steroids + prolactin (1 i.u.) |
|---|---|---|---|---|
| McG | 26 | 11±5 (1/10) | 31±14 (5/10) | 61±13 (1/10) |
| Col | 31 | | 45±17** (1/6) | |
| Mi | 38 | | 35±6** (2/10) | |
| deS | 19 | 5±2 (4/10) | 21±9* (1/6) | 36±7 (3/10) |
| So | 11 | | 14±3** (1/6) | |
| Li | 38 | | 31±4* (3/8) | |
| Qu | 15 | | 37±13*** (7/10) | |
| Number of mice | | 15 | 36 | 16 |
| Overall mean | | 9±3 | 30±4*** | 50±8 |
| LF | Not pregnant | 10±4 (0/10) | 29±9** (9/13) | 43±6 (0/10) |
| FC | | | 25±3** (6/12) | |
| JC | | | 32±6*** (2/9) | |
| Number of mice | | 10 | 17 | .. |
| Overall mean | | 10±4 | 29±3*** | .. |

* Significantly different from response to steroids alone ($P <$ 0·1*, $<$ 0·05**, $<$ 0·01***).

Six of the 7 samples from pregnant women that were tested and all 3 of the samples from non-pregnant women significantly increased the club count (Table II).

### DISCUSSION

*Mammotrophic activity* is used here in a limited and purely empirical sense: it refers to the capacity to increase the club count in the mammary glands of hypophysectomized weanling male mice of a particular strain when the mice are treated with oestrone and progesterone in addition to the material under test.

Our results confirm the earlier evidence (Hadfield, 1957 ; Hadfield and Young, 1958) that such activity is a property of prolactin and of growth hormone and that it is present in the urine and serum from normal women. Not unexpectedly, we find it is also present in the serum from pregnant women.

The activity found in normal serum would represent about 0·5–1 i.u. of prolactin or 0·05–0·1 mg. of growth hormone per ml. Current estimates suggest that normal blood contains 0·002–0·2 i.u. of prolactin (Simkin and Goodart, 1960) and 0·2 $\mu$g. of growth hormone (Read and Bryan, 1960) per ml. Thus it is unlikely that either hormone is alone responsible for the mammotrophic activity in serum. It is much more probable that the activity represents the resultant effect of both hormones and possibly of those other hormones that are concerned in mammary development in rats and mice (Lyons, 1958 ; Nandi, 1959).

The results in Table II show that the method we have used is adequate for demonstrating the presence of mammotrophic activity in serum. It is not so satisfactory when applied to neat urine though when the reduced sensitivity of the mice is taken into consideration we regard the three positive results in Table I

FIG. 1.—Dose : response lines for prolactin (●—●) and growth hormone (○—○) in 1959 and 1960.

as sufficient confirmation of the more convincing evidence previously obtained (Hadfield, 1957 ; Hadfield and Young, 1958). It seems that the activity in urine must be concentrated if it is to be demonstrated with any regularity. The dose: response relations for prolactin were very similar to those obtained in a previous study (Young and Fraser, 1960) using the original steroid dosage but the two sets of results with growth hormone did not agree so well (Fig. 1). It may be noticed that both 1959 graphs flatten off at a count of about 50–70 clubs per mouse and there has been much evidence of a similar plateau in the present experiments with prolactin (see Appendix). This may indicate that the mammary response is biphasic.

Despite simplification of the injection procedure, the modified club-count assay described in the Appendix is laborious and the variability of the mammary response from mouse to mouse severely limits the accuracy with which mammotrophic activity can be measured. Taken over several experiments the mean square error of the mammary response is about 1200, and the slope of the dose : response curve 20. If the strength of the test preparation is the same as the standard, the error of the log potency ratio (M), which is equivalent to the percentage error of the actual potency ratio, is roughly

$$\text{S.E. (M)} = \frac{s}{b}\sqrt{\frac{2}{\text{N}}}$$

where $s^2$ is the mean square error of the response, $b$ the slope and N is the number of animals. Inserting the above values for $s$ and $b$ and correcting to natural logs it is found that about 120 mice are needed to give 25 per cent accuracy in the potency ratio. On the whole this is an optimistic estimate, and if the unknown preparation differed considerably from the standard, the accuracy would be much lower. On the other hand, the mean square error in some cases may be as low as 600. However there is little doubt that consistently reliable results could only be obtained by using much larger numbers of animals than is feasible in practice.

It may be that treatment with other hormones concerned in mammary development in addition to oestrone and progesterone would increase the sensitivity of the hypophysectomized mice or reduce the variability of the response. On the other hand, such a procedure might offset one of the advantages of the biological method in studies of mammary cancer ; its measurement of an effective activity that may be the resultant of the action of several hormones. Only further studies can decide this question or whether an alternative and more practicable method of assay can be devised.

### SUMMARY

Mammotrophic activity was assessed by injecting material to be tested into hypophysectomized weanling male mice of the A2G strain that were being injected at the same time with oestrone and progesterone and counting the increase in the number of swollen end-buds (clubs) of the mammary ducts.

Omitting samples that killed all, or all but one, of the test animals, activity was demonstrated in 3 out of 11 samples of urine, and in all of 3 samples of serum, from normal young women. Six out of 7 samples of serum from pregnant women were also active. No activity was demonstrable in 4 samples of urine from women past the menopause.

Some simplification of the method of testing was effected without reducing the variability of the response. This variability renders the method unsuitable for quantitative assays at present.

We are very grateful to the Endocrine Study Section, U.S.A. National Institutes of Health for generous gifts of prolactin and growth hormone, to Professor N. F. Morris, Charing Cross Hospital Medical School, for supplying the sera from pregnant women, and to Mr. John Gilbert and Mr. Michael Hart for much painstaking and assiduous technical assistance.

### REFERENCES

HADFIELD, G.—(1957) *Lancet*, i, 1058.
*Idem* AND YOUNG, STRETTON—(1956*a*) *Brit. J. Cancer*, **10**, 145.—(1956*b*) *Ibid.*, **10**, 324.—(1958) *Lancet*, i, 568.
LYONS, W. M.—(1958) *Proc. Roy. Soc.* B, **149**, 303.
NANDI, S.—(1959) *Univ. Calif. Publ. Zool.*, **65**, 10.
READ, C. H. AND BRYAN, G. T.—(1960) *Ciba Found. Coll. Endocr.*, **13**, 68.
SIMKIN, B. AND GOODART, D.—(1960) *J. clin. Endocr.*, **20**, 1095.
YOUNG, STRETTON—(1957) *Brit. J. Cancer*, **11**, 116.
*Idem* AND FRASER, L. E.—(1960) *Ibid.*, **14**, 285.

### APPENDIX

### *Attempted Improvement of the Assay*

*Steroid dosage*

The doses of oestrone and progesterone (1 μg. and 5 mg.) used routinely seemed unnecessarily large. Reduction of the total doses to 0·1 μg. of oestrone and 1 mg. of progesterone did not influence the club count produced in response to 5 i.u. of prolactin. The same club count was also induced by 1 i.u. of prolactin suggesting that a plateau had been attained in the dose : response curve (Table III, Experiment A).

*Steroid solvents*

If lower doses of the two steroids were to be used then it should be possible to substitute a more convenient solvent than arachis oil, which is inconvenient to handle, persists at the injection site, and often interferes with the clean dissection of the mammary glands from the pelt.

Fifty per cent aqueous propylene glycol proved an unsatisfactory solvent. Responses were usually reduced in comparison with tests using arachis-oil solutions and the dose given (0·1 ml. twice daily) was on the borderline of toxicity for larger

TABLE III.—*Modification of Assay Procedure*

| Experiment | Steroid solvent | Progesterone Dose (mg.) | Progesterone Number of injections | Oestrone (total dose μg.) | Prolactin (total dose i.u.) | Number of mice | Club count ± s.e.$_m$ |
|---|---|---|---|---|---|---|---|
| A | Arachis oil | 5·0 | 10 | 1·0 | 5·0 | 30 | 64±6 |
| | | 1·0 | 10 | 0·1 | 5·0 | 30 | 72±6 |
| | | 1·0 | 10 | 0·1 | 1·0 | 27 | 68±5 |
| B | Water | 1·0 | 1 | 0·1 | 5·0 | 10 | 78±10 |
| | | 1·0 | 10 | 0·1 | 5·0 | 8 | 71±8 |
| | | 2·0 | 1 | 0·1 | 5·0 | 10 | 80±10 |
| | | 2·0 | 10 | 0·1 | 5·0 | 10 | 71±2 |
| C | Water | 1·0 | 10 | 0·1† | 5·0 | 13 | 61±9 |
| | | 1·0 | 10 | 0·1* | 5·0 | 18 | 57±6 |
| | | 2·0 | 10 | 0·1† | 5·0 | 8 | 66±9 |
| | | 2·0 | 10 | 0·1* | 5·0 | 10 | 71±2 |

† Oestrone and progesterone given together as mixed solution.
* Oestrone and prolactin given together as mixed solution.

volumes or increased concentration of the solution killed some, or all, of the mice injected.

The use of aqueous solutions or suspensions, however, had several advantages. An aqueous suspension of progesterone given as a single subcutaneous injection, was as effective as the same dose given in 10 injections (Table III, Experiments A and B) and aqueous solutions of oestrone could be mixed with the prolactin solution and injected in combination without affecting the response (Table III, Experiment C). This procedure not only reduced the labour of multiple injections but also eased the dissection of the pelts and was therefore adopted in all subsequent tests. With this simpler procedure it was possible to undertake a factorial experiment examining the change in response with variations in dosage of all three hormones.

*Factorial experiment on hormone dosage*

A 3 × 3 × 4 factorial experiment was set up with 3 doses of oestrone (0·05, 0·1 and 0·2 μg.), 3 doses of progesterone (0·5, 1 and 2 mg.) and 4 doses of prolactin (0, 0·5, 1 and 2 i.u.). There were originally 4 mice in each of the 27 groups receiving prolactin and 2 mice in each of the 9 groups receiving no prolactin. Since the different steroid doses had little, if any, effect on the response to prolactin, no elaborate statistical formulation need be given. In Table IV the full data are arranged with reference to prolactin dosage, first without regard to steroid dosage and then with regard to oestrone dosage and progesterone dosage separately. Clearly prolactin dosage within the range 0·5–2·0 i.u. had little if any effect on response and this is true whatever the steroid dosage within the range of 0·05–0·2 μg. of oestrone and 0·5–2 mg. of progesterone.

TABLE IV.—*Club Counts* ± $s.e._m$ *in Factorial Experiment on Steroid Dosage*

| | Dose of prolactin in i.u. (total number of mice) | | | |
|---|---|---|---|---|
| Analysis | 0·0 (16) | 0·5 (34) | 1·0 (33) | 2·0 (32) |
| All mice . | 10±2 | 46±4 | 51±6 | 56±5 |
| Oestrone | | | | |
| 0·05 μg. . | 12±6 | 50±8 | 42±7 | 61±9 |
| 0·10 μg. . | 8±4 | 47±6 | 61±11 | 51±10 |
| 0·20 μg. . | 10±2 | 42±8 | 50±9 | 54±9 |
| Progesterone | | | | |
| 0·5 mg. . | 13±5 | 42±8 | 36±4 | 51±9 |
| 1·0 mg. . | 10±5 | 46±6 | 64±16 | 60±9 |
| 2·0 mg. . | 8±5 | 52±8 | 55±10 | 56±9 |

*Dose : response relation for prolactin and growth hormone*

Since the variation in steroid dosages used in the previous experiment had no significant effect on the results, the median doses (1 mg. of progesterone suspension and 10 × 0·01 μg. of oestrone in aqueous solution) were used in assays of the mammotrophic activity of prolactin and growth hormone. The results (Table V) show clearly how individual variation in response could vitiate conclusions based on small groups of animals or small differences in dose. With 10 mice per group,

TABLE V.—*Responses to Prolactin or Growth Hormone given with 1 mg. Progesterone and* 0·1 *μg. Oestrone*

| Prolactin | | Growth hormone | |
|---|---|---|---|
| Dose (i.u.) | Club count + s.e.$_m$ | Dose (mg.) | Club count ± s.e.$_m$ |
| 0 | 18±4 | 0 | 7±4 |
| 0·12 | 45±5 | 0·03 | 32±10 |
| 0·25 | 36±9 | 0·06 | 24±10 |
| 0·50 | 40±10 | 0·12 | 46±15 |
| 1·00 | 72±10 | 0·25 | 53±12 |
| 2·00 | 60±8 | 0·50 | 25±4 |

a lower average response can be elicited by a 4-fold increase in the dose of prolactin (compare the responses to 0·12 i.u. and 0·50 i.u.) and a reduction in response on doubling the dose is common (cf. 0·12–0·25 i.u., 1·0–2·0 i.u.). Despite this variation a significant regression line is achieved ($b = 22 \pm 8{\cdot}8$, $t = 2{\cdot}5$, 12 D.F., $P < 0{\cdot}05$). With growth hormone, however, a very low average response to the highest dose made the regression line insignificantly different from horizontal ($b = 9{\cdot}5 \pm 14{\cdot}7$, $t = 0{\cdot}65$, 29 D.F., $P < 0{\cdot}05$).